# Engineering 2D Materials from Single‐Layer NbS_2_

**DOI:** 10.1002/smll.202408044

**Published:** 2024-11-25

**Authors:** Timo Knispel, Daniela Mohrenstecher, Carsten Speckmann, Affan Safeer, Camiel van Efferen, Virgínia Boix, Alexander Grüneis, Wouter Jolie, Alexei Preobrajenski, Jan Knudsen, Nicolae Atodiresei, Thomas Michely, Jeison Fischer

**Affiliations:** ^1^ II. Physikalisches Institut Universität zu Köln Zülpicher Straße 77 D‐50937 Köln Germany; ^2^ NanoLund and Division of Synchrotron Radiation Research, Department of Physics Lund University Lund SE‐221 00 Sweden; ^3^ MAX IV Laboratory Lund University Lund SE‐221 00 Sweden; ^4^ Peter Grünberg Institut (PGI‐1) Forschungszentrum Jülich Wilhelm‐Johnen‐Straße D‐52428 Jülich Germany

**Keywords:** covalent transformation, molecular beam‐epitaxy, niobium disulfide, single layer

## Abstract

Starting from a single layer of NbS_2_ grown on graphene by molecular beam epitaxy, the single unit cell thick 2D materials Nb_5/3_S_3_‐2D and Nb_2_S_3_‐2D are created using two different pathways. Either annealing under sulfur‐deficient conditions at progressively higher temperatures or deposition of increasing amounts of Nb at elevated temperature result in phase‐pure Nb_5/3_S_3_‐2D followed by Nb_2_S_3_‐2D. The materials are characterized by scanning tunneling microscopy, scanning tunneling spectroscopy, and X‐ray photoemission spectroscopy. The experimental assessment combined with systematic density functional theory calculations reveals their structure. The 2D materials are covalently bound without any van der Waals gap. Their stacking sequence and structure are at variance with expectations based on corresponding bulk materials highlighting the importance of surface and interface effects in structure formation.

## Graphical abstract

Starting from a single‐layer NbS_2_ on graphene, new 2D materials Nb_5/3_S_3_‐2D and Nb_2_S_3_‐2D are synthesized via sulfur‐deficient annealing or Nb deposition at elevated temperatures. These covalently bound materials exhibit unique structures influenced by surface effects, differing from expected bulk stacking sequences.

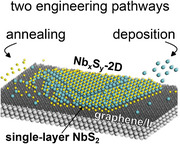

## 1 Introduction

Thinning down a layered material to a few or single layers transforms it into a 2D material. The typical way of obtaining a 2D material is by exfoliation of the bulk crystal. The method is simple, the structural quality of exfoliated layers is generally very good,^[^ ^1^, ^2^ ^]^ stacking of layers to create vertical heterostrutures with new functions is straightforward,^[^ ^3^, ^4^ ^]^ and finally twisted stacking opened the door for moiré physics.^[^ ^5^ ^]^

Nevertheless, exfoliation as a method has several significant limitations. Beyond its fundamental scalability issues, exfoliation is ineffective in preparing single or few‐layer thick 2D materials from covalently bound bulk crystals. Such crystals lack van der Waals gaps and, consequently, cannot be adequately exfoliated. Additionally, exfoliation cannot be used for synthetically constructed 2D materials that have no bulk counterparts in terms of structure or composition.

The scope of 2D materials can be substantially broadened by the use of growth methods like molecular beam epitaxy (MBE) or chemical vapor deposition (CVD). For example, provision of more than one metal during growth enables one to explore the entire composition space between two dissimilar transition metal dichalcogenides (TMDCs) on the single layer level^[^ ^6^ ^]^ or to create vertical TMDC heterostructures with continuously tunable moiré periodicity by using the composition dependent lattice parameters.^[^ ^7^ ^]^ Variation of the metal chemical potential enables the production of thin films across the entire sequence of self‐intercalation compounds known from bulk crystals, allowing for the discovery of magnetic order in some of these intercalated phases.^[^ ^8^ ^]^

Annealing an initial transition metal chalcogenide with or without chalcogene flux, possibly following prior metal deposition, is another strategy applied to create new phases. Examples are the annealing‐induced single‐layer transformations of CrSe_2_ into Cr_2_Se_3_,^[^ ^9^ ^]^ of VS_2_ into stripped V_2_S_3_^[^ ^10^ ^]^ or V_4_S_7_,^[^ ^11^ ^]^ of PtTe_2_ into Pt_2_Te_2_,^[^ ^12^ ^]^ of α‐FeSe into kagome Fe_5_S_8_,^[^ ^13^ ^]^ or of Bi_2_Se_3_ into MnBi_3_Se_4_.^[^ ^14^ ^]^ The enumeration is by far not complete.

In the present manuscript, we investigate phase transitions of single‐layer NbS_2_. This TMDC has attracted substantial research interest due to its superconductivity in the bulk^[^ ^15^, ^16^, ^17^ ^]^ and its charge‐density wave in the single layer.^[^ ^18^, ^19^ ^]^ Moreover, self‐intercalated Nb_1 + x_S_2_ is an excellent catalyst for the hydrogen evolution reaction.^[^ ^20^ ^]^

Besides the van der Waals material NbS_2_, the Nb‐S phase diagram displays a zoo of phases without van der Waals gap, i.e., being covalently bound, of which the structures were carefully investigated by X‐ray diffraction.^[^ ^21^, ^22^ ^]^ Whether any of these covalently bound bulk phases possesses a 2D pendant is still unexplored. Here we establish and characterize two Nb_*x*_S_*y*_‐2D compounds of single‐unit cell thickness, namely Nb_5/3_S_3_‐2D and Nb_2_S_3_‐2D. To avoid confusion with bulk materials with the same composition, but different structure, “‐2D” is attached to the stoichiometric formulas indicating the yet undescribed 2D materials. Due to their single‐unit cell thickness, these compounds are referred to as single‐layer materials. A single layer consists of stacked S–Nb–S–Nb–S planes of atoms. Starting from MBE‐grown single‐layer NbS_2_, the new phases are established under ultrahigh vacuum conditions via two different kinetic pathways, either through pure annealing or by metal deposition at elevated temperature. We find that each phase can be prepared phase‐pure, making its investigation by averaging techniques feasible.

Beyond our methodology for creating these materials, we highlight three important findings that are of broader relevance and can be generalized to the covalent growth of other layered 2D materials. First, applying careful titration based on a well‐calibrated evaporator is an efficient tool to determine the stoichiometry of an unknown compound resulting from phase transformation. Second, the structures of the resulting 2D materials differ from the known bulk phases, although their chemical composition is rather similar. Our results, thus indicate that surface effects are important and consequently assumptions that the compounds can be described from corresponding bulk phases may fail. Third, we show that the explicit inclusion of the substrate in theoretical calculations is necessary to provide a valuable insight into the range of possible phases and to guide the interpretation of experiments.

## 2 Results

### 2.1 Concepts for Covalent Transformation of Single‐Layer NbS_2_

**Figure 1:**
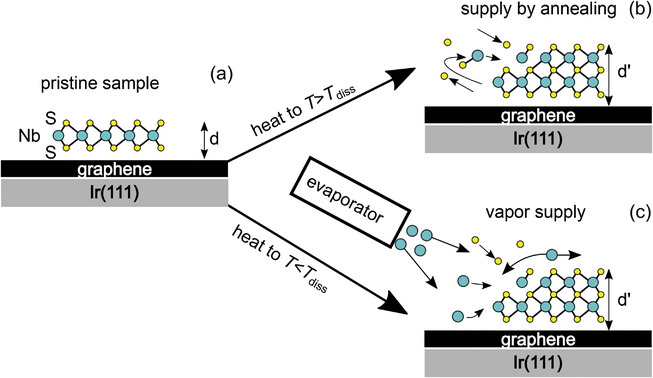
Concept of covalent transformation. a) Single‐layer H‐NbS_2_ on Gr/Ir(111). b) Covalent transformation by heating and dissociation. c) Covalent transformation by deposition of additional Nb.

Our course of action to achieve covalent growth is exemplified for NbS_2_ in **Figure** **1**. Figure 1a displays single‐layer H‐NbS_2_ grown on graphene (Gr) on Ir(111). The first strategy is to heat up the sample to a temperature *T*_diss_ that causes NbS_2_ to partially dissociate (Figure 1b). Some of the S of NbS_2_ escapes into vacuum or intercalates between Gr and Ir(111). The remaining Nb excess triggers a phase transformation to a covalently bonded niobium‐rich compound composed of 3 atomic planes of S separated by Nb planes. The new compound exhibits an increased height *d*′. Evidently, the partial dissociation of the single‐layer NbS_2_ of height *d* in combination with the larger height *d*′ of the new phase formed without supply of additional material causes a reduction in sample coverage. The second strategy is to induce covalent growth by deposition of additional Nb at a temperature *T* < *T*_*diss*_, see Figure 1c. When arriving on the surface, the deposited Nb reacts with the existing NbS_2_ and thereby triggers the phase transformation. In both cases, identical phases can be obtained.

### 2.2 Covalent Transformation by NbS_2_ Annealing

This section describes the transformation of NbS_2_ into two different phases richer in Nb obtained by heating to successively higher temperatures with *T* > *T*_*diss*_.

**Figure 2:**
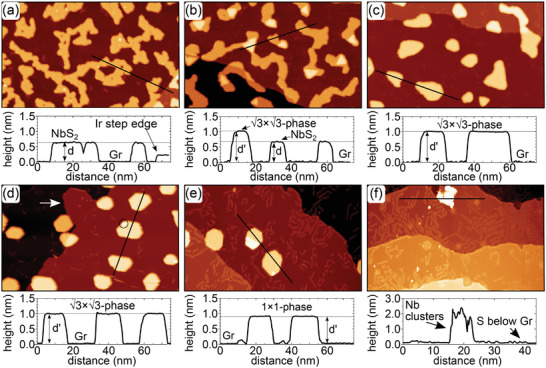
STM topographs of an isochronal annealing sequence of initial single‐layer NbS_2_ islands without supply of additional S. Annealing time intervals are 360 s. a) Single‐layer NbS_2_ islands after room temperature growth and annealing to 820 K. b–f) After additional annealing to (b) 920 K, (c) 1020 K, (d) 1120 K, (e) 1220 K, and (f) 1320 K. In (d) a bright spot at an island edge is encircled and a peninsula attached to an edge is highlighted by a white arrow. Height profiles along the black lines are shown below the topographs. Height levels *d* = 0.62 nm, *d*′ = 0.99 nm and *d*′ = 0.93 nm distinguish between single‐layer NbS_2_, $\sqrt{3}\times \sqrt{3}$ ‐ phase, 1 × 1 ‐ phase, respectively. Image information: for all size is 150 nm × 90 nm, (a) *V*_s_ =1.0 V, *I*_t_ = 0.23 nA; (b) *V*_s_ = 0.95 V, *I*_t_ = 0.34 nA; (c) *V*_s_ = 1.0 V, *I*_t_ = 0.26 nA; (d) *V*_s_ = 0.92 V, *I*_t_ = 0.33 nA; (e) *V*_s_ = 1.0 V, *I*_t_ = 0.32 nA; (f) *V*_s_ = 2.2 V, *I*_t_ = 0.06 nA. In (f) a large tunneling resistance was chosen, to avoid tip sample interaction with the tall cluster.

**Figure** **2**a displays a scanning tunneling microscopy (STM) image of pristine single‐layer NbS_2_ islands grown on Gr/Ir(111) by room temperature deposition of 0.34 ML Nb in S vapor and subsequent annealing to 820 K (compare Methods). The islands cover an area fraction of 0.34, are continuous over Ir substrate steps under the Gr carpet, and are of irregular shape. The islands display an apparent height of *d* = 0.62 ± 0.01 nm at *V*_s_ = 1.00 V as exemplified by the height profile below the topograph (*d* depends slightly on the tunneling voltage; *d* = 0.58 ± 0.01 nm at *V*_s_ = −1.00 V). The measured apparent heights fit reasonably well to the apparent height of 0.578 nm reported for single‐layer NbS_2_ on Gr/6H‐SiC(0001)^[^ ^18^ ^]^ and to half of the c‐axis lattice constant of 1.195 nm of bulk NbS_2_.^[^ ^16^ ^]^ In our previous work,^[^ ^19^ ^]^ it was firmly established that under these conditions NbS_2_ on Gr/Ir(111) grows in the H‐phase, in agreement with the findings for single‐layer NbS_2_ on Au(111)^[^ ^23^ ^]^ and bulk NbS_2_.^[^ ^16^ ^]^

While NbS_2_ islands are stable at 820 K independent of the duration of annealing, after annealing the sample to 920 K, the island area fraction decreases to 0.29. Higher triangular‐shaped areas emerge within the NbS_2_ islands Figure 2b). In these areas, height profiles give an increased apparent height of *d*′ = 0.99 nm at *V*_s_ = 1.00 V (*d*′ = 0.90 nm at *V*_s_ = −1.00 V). This height is inconsistent with bilayer NbS_2_, which has an apparent height of 1.22 nm at *V*_s_ = 1.00 V, as displayed in Figure S1 (Supporting Information). These higher areas are designated as in the $\sqrt{3}\times \sqrt{3}$ ‐ phase, since below it will be shown that they exhibit a $\left(\sqrt{3}\times \sqrt{3}\right)R30^{\circ }$ superstructure and are of composition Nb_5/3_S_3_‐2D.

After annealing the sample to 1020 K (Figure 2c), all islands display an apparent height of *d*′ = 0.99 nm, consistent with the assumption that the islands have entirely transformed to the $\sqrt{3}\times \sqrt{3}$ ‐ phase, indicating phase purity. The island area fraction decreased further to 0.17.

At first glance, annealing the sample to 1120 K as shown in Figure 2d does not change the situation. From the height profile, all islands still display a height *d*′ = 0.99 nm characteristic of the $\sqrt{3}\times \sqrt{3}$ ‐ phase. The islands shape is more regular, mostly hexagonal. The island area fraction further decreases to 0.12. On the island edges a few tiny bright spots appear (one is encircled in Figure 2d) which could be due to metallic Nb resulting from Nb_*x*_S_*y*_ dissociation and S loss. A peninsula attached to an Ir substrate step is also visible and highlighted with a white arrow. From its different contrast (for a contrast enhanced image see Figure S2, Supporting Information), we tentatively conclude that it is formed by intercalated Nb. It is well known that metal on Gr on a metal substrate eventually intercalates. The driving force is the strong adhesion of the metal–metal bonds. The pathway of metal intercalation is less clear, but intercalation at wrinkles, at point defects and by self‐etching were proposed.^[^ ^24^, ^25^, ^26^ ^]^

The $\sqrt{3}\times \sqrt{3}$ ‐ phase islands are easy to shift laterally as a whole by the STM tip (see Figure S3a, Supporting Information) consistent with being physisorbed to Gr. The liberated Gr, initially under an island, displays neither structural nor height changes. Thereby, it is ruled out that the height increase to *d*′ = 0.99 nm is caused by changes in the Gr height level, e.g., by intercalation of S or Nb underneath the islands.

After annealing to 1220 K (Figure 2e) the island area fraction is further reduced to 0.10. Two different height levels are present, neither of which coincides with the aforementioned ones. As obvious from the height profile, the dominant height level is *d*′ = 0.93 nm at *V*_s_ = 1.00 V (*d*′ = 0.94 nm at *V*_s_ = −1.00), slightly, but clearly, lower than the $\sqrt{3}\times \sqrt{3}$ ‐ phase height. We designate areas with this height level as 1 × 1 ‐ phase, since below it will be shown that they exhibit no superstructure and are of composition Nb_2_S_3_‐2D. Besides 1 × 1 ‐ phase islands, stripes and patches with an apparent height of ≈0.1 nm with respect to the Gr base level are prominent now. A few of these stripes are already present at lower annealing temperature (compare Figure 2d). Such stripes are well known also from other metal sulfide growth experiments on Gr/Ir(111) (see ref. [27] and Supporting Information of ref. [19]) and were assigned to S that intercalated under Gr and adsorbed to Ir(111) where it forms a $\left(\sqrt{3}\times \sqrt{3}\right)R30^{\circ }$ adsorbate layer with respect to the Ir(111) surface lattice.^[^ ^28^ ^]^ This $\left(\sqrt{3}\times \sqrt{3}\right)R30^{\circ }$ superstructure of S is unrelated to the $\sqrt{3}\times \sqrt{3}$ ‐ phase, since the latter is with respect to the NbS_2_ lattice, and not with respect to Ir(111). The intercalated S may stem from the phase transformation of the $\sqrt{3}\times \sqrt{3}$ ‐ phase into the 1 × 1 ‐ phase, which would indicate a change toward a less S‐rich stoichiometry of 1 × 1 ‐ phase. However, also mere decomposition of islands would release S.

Up to 1220 K, the annealing sequence was repeated for several samples yielding the same results (compare Figure S4, Supporting Information).

After the final annealing step to 1320 K (compare Figure 2f) even a large area search by STM does not show any Nb–S islands. The complete decomposition of Nb–S islands has to be concluded. What remains is intercalated S (well visible as ≈0.1 nm high stripes and islands), local modifications at step edges that we tentatively attribute to be a consequence of Nb intercalation, and rare nm‐tall clusters. Figure 2f was chosen to show one of these. We tentatively assume that these rare clusters are linked to residual non‐intercalated Nb.

Our X‐ray photoemission spectroscopy (XPS) measurements, discussed below, indicate that even after annealing to 1320 K, a substantial amount of Nb remains on the surface in a chemical state distinct from that of Nb–S compounds (see Figure S5, Supporting Information). Since the intensity cannot be fully attributed to the rare clusters, we conclude that Nb is dispersed across the Ir(111) surface.

**Figure 3:**
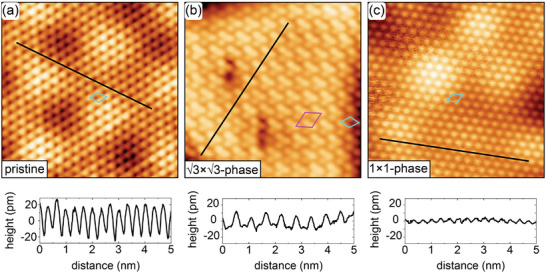
Atomic resolution STM topographs of a) pristine single‐layer NbS_2_, b) the $\sqrt{3}\times \sqrt{3}$ ‐ phase, and c) the 1 × 1 ‐ phase taken at 1.7 K. In the STM topographs the unit cells of the three phases are indicated by cyan rhomboids. Magenta rhomboid is the $\left(\sqrt{3}\times \sqrt{3}\right)R30^{\circ }$ superstructure. Height profiles along the black lines are shown below the topographs. Image information: for all size 6 nm × 6 nm and *T*_s_ = 1.7 K, (a) *V*_s_ = 50 mV, *I*_t_ = 0.5 nA; (b) *V*_s_ = 100 mV, *I*_t_ = 0.80 nA; (c) *V*_s_ = 100 mV, *I*_t_ = 0.70 nA.

To justify the designation of the 0.99 or 0.93 nm high islands as being $\sqrt{3}\times \sqrt{3}$ ‐ phase or 1 × 1 ‐ phase, we present atomically resolved STM images of the island structures from Figure 2a,c,e. For reference, **Figure** **3**a displays atomically resolved NbS_2_. It has a lattice parameter of 0.331(3) nm as established in our previous work,^[^ ^19^ ^]^ in good agreement with values found for the single‐layer NbS_2_ on bilayer Gr/6H‐SiC(0001) (0.334 nm)^[^ ^18^ ^]^ and bulk 2H‐NbS_2_ (0.3324 nm).^[^ ^16^ ^]^ The atomic corrugation is on the order of 35 pm as apparent from the height profile along the black line shown below the topograph. The height modulation on a length scale of ≈2.5 nm is due to the Gr/Ir(111) moiré pattern imposed on NbS_2_.^[^ ^19^, ^27^ ^]^

An atomically resolved topograph of the $\sqrt{3}\times \sqrt{3}$ ‐ phase is shown in Figure 3b. It displays a clear $\left(\sqrt{3}\times \sqrt{3}\right)R30^{\circ }$ superstructure with respect to the original NbS_2_ lattice. Since all atoms in the top layer can still be recognized, the superstructure results from a trimerization of the S atoms. At the island edges the trimerization seems to fade away. The superstructure is associated with a height modulation on the order of 15 pm. The superstructure displays a lattice size of 0.577 ± 0.05 nm, which corresponds to $\sqrt{3}a$, with *a* = 0.333 ± 0.03 nm. Low‐energy electron diffraction (LEED) obtained after annealing to 1020 K displays faint spots of a $\left(\sqrt{3}\times \sqrt{3}\right)R30^{\circ }$ superstructure with respect to NbS_2_ (Figure S6, Supporting Information].

Figure 3c shows an atomically resolved topograph of the 1 × 1 ‐ phase. It features a 1 × 1 structure with the lattice parameter *a* = 0.330 ± 0.05 nm, identical to the one of single‐layer NbS_2_ within the limits of error. The 1 × 1 ‐ phase is distinct from pristine NbS_2_ because: i) it differs in height (0.93 nm vs 0.62 nm); ii) the atomic corrugation is only in the order of 5 pm, reduced by about a factor of 7 compared to the pristine NbS_2_; and iii) the Gr/Ir(111) moiré corrugation is shining through the 1 × 1 ‐ phase islands is considerably damped compared to NbS_2_.

**Figure 4:**
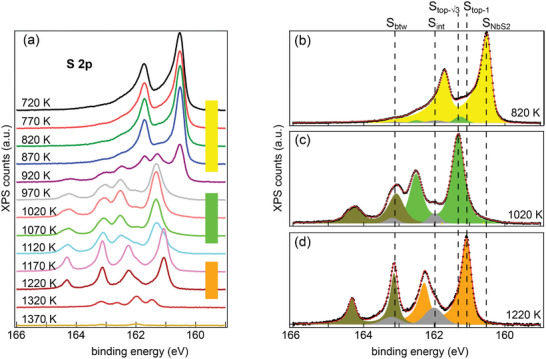
a) XPS of the S 2p core level of initial single‐layer NbS_2_ on Gr/Ir(111) transformed during annealing. After room temperature growth, for each spectrum the sample was annealed to the indicated temperature without supply of additional S and cooled down to 300 K for measurements. The spectra are grouped in three temperature ranges according to their similarities: yellow, green, and orange. b–d) S 2p core level spectra after annealing to (b) 820 K, (c) 1020 K, and (d) 1220 K fitted with components.

In order to obtain complementary chemical information about the annealing‐induced phases, XPS of the S 2p, Nb 3d, C 1s, and Ir 4f core levels was performed. **Figure** **4**a shows the S 2p core‐level spectra of samples with increasing annealing temperatures from 720 K (top) to 1370 K (bottom). The S 2p core level is spin‐orbit split into a 2p_3/2_ and 2p_1/2_ doublet with an energy separation of 1.19(3) eV. The S 2p components are referenced in the following to the lower binding energy 2p_3/2_ peak. The appearance of the spectra has three distinct temperature ranges: i) 720 K up to 870 K (yellow bar), ii) 970 to 1070 K (green bar), and iii) 1170 to 1220 K (orange bar). These temperature ranges agree well with the temperature ranges of the phases identified in STM (compare Figure 2). The distinct spectra for each temperature range is evidence of phase purity. The sequence of spectra shows a decrease in S 2p intensity with temperature (see also Figure S7, Supporting Information), and after annealing at 1320 K the S 2p signal has nearly vanished consistent with the decomposition of Nb–S compounds and subsequent S desorption. Spectra for Nb 3d, C 1s, and Ir 4f core‐levels are displayed in Figures S5 and S8 (Supporting Information).

The spectra in Figure 4b–d correspond to single‐layer NbS_2_, $\sqrt{3}\times \sqrt{3}$ ‐ phase, and 1 × 1 ‐ phase, respectively. The S 2p spectrum of NbS_2_ in Figure 4b displays mainly a single spin‐orbit doublet (yellow), designated S_NbS2_ and located at 160.60(0) eV. The S_NbS2_ component is attributed to top and bottom S in NbS_2_. In the $\sqrt{3}\times \sqrt{3}$ ‐ phase spectrum after annealing to 1020 K in Figure 4c the S_NbS2_ component is absent and two new main components are present: $S_{\mathrm{top}-\sqrt{3}}$ at 161.33 eV (green) and S_btw_ at 163.08 eV (olive).

The substantial core level shifts are consistent with a phase transformation from NbS_2_ to the $\sqrt{3}\times \sqrt{3}$ ‐ phase. Having the largest intensity, the $S_{\mathrm{top}-\sqrt{3}}$ component is associated with the top sulfur layer. The S_btw_ component (olive) with a significant core level shift of 2.48 eV compared to S_NbS2_ is tentatively assigned to sulfur in a lower atomic plane (due to its lower intensity) and in a very different chemical environment than in NbS_2_. The origin of the S_btw_ will be clarified further below with the help of additional information from STM and density functional theory (DFT). The gray *S*_int_ component at 161.99 eV is attributed to intercalated S lost during the phase transformation. The same S 2p component growing in intensity during annealing has been found for VS_2_.^[^ ^11^ ^]^

The 1 × 1 ‐ phase spectrum after annealing to 1220 K in Figure 4d retains the S_btw_ component at 163.13 eV (olive) and develops a new S_top‐1_ component at 161.05 eV (orange), shifted by 0.28 eV with respect to the $S_{\mathrm{top}-\sqrt{3}}$ component. Overall, the spectrum is quite similar to the $\sqrt{3}\times \sqrt{3}$ ‐ phase spectrum and distinct from the NbS_2_‐spectrum. Remarkably, the S_top‐1_ and S_btw_ components are sharp with full width at half maximum (FWHM) of 0.29 and 0.27 eV, almost halved compared to the $\sqrt{3}\times \sqrt{3}$ ‐ phase. The narrow peaks indicate the homogeneous state of the S in this phase. The S_int_ component is further increased due to the release of sulfur during the transformation from the $\sqrt{3}\times \sqrt{3}$ ‐ phase to the 1 × 1 ‐ phase.

Inspection of the Nb 3d core level spectra reveals the same three distinct temperature ranges for single‐layer NbS_2_, $\sqrt{3}\times \sqrt{3}$ ‐ phase, and 1 × 1 ‐ phase (compare Figure S5, Supporting Information).

### 2.3 Covalent Transformation of NbS_2_ by Nb Vapor Supply

The phase transformations of NbS_2_ are likely to be triggered by Nb excess resulting from the loss of S due to annealing. If this rationale is correct, one could expect the transformation also to take place already at temperatures below *T*_diss_, if additional Nb is supplied. Moreover, by controlling the amount of Nb supplied, it might be possible to select the resulting phase.

**Figure 5:**
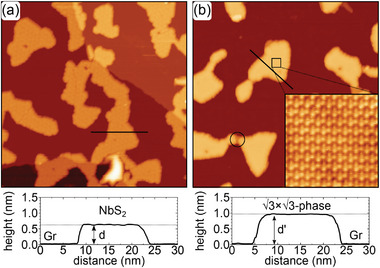
Formation of the $\sqrt{3}\times \sqrt{3}$ ‐ phase by Nb supply. a) Single‐layer NbS_2_ grown by deposition of 0.36 ML Nb in S background pressure at room temperature and annealed to 820 K in the absence of additional S supply. The bright protrusion at the bottom of the image consists of an Ar‐filled Gr‐blister.^[^ ^46^ ^]^ b) Sample after deposition of additional 0.12 ML Nb at 820 K in the absence of additional S supply results in the formation of the $\sqrt{3}\times \sqrt{3}$ ‐ phase. A tiny piece of single‐layer NbS_2_  is encircled. Inset: atomic resolution topograph of boxed area. Height profiles are taken along the black lines in the STM topographs. Image information: (a) size 100 nm × 100 nm, *V*_s_ = 1.0 V, *I*_t_ = 1.00 nA; (b) size 100 nm × 100 nm, *V*_s_ = 1.0 V, *I*_t_ = 1.0 nA; Inset: 5 nm × 5 nm, *V*_s_ = 0.1 V, *I*_t_ = 5 nA.

To test this idea, 0.12 ML Nb was deposited at 820 K on pre‐grown single‐layer NbS_2_ islands with an area fraction of 0.36 (**Figure** **5**a). Plain annealing at 820 K neither causes NbS_2_ dissociation nor changes the coverage fraction. Upon deposition of Nb, the single‐layer NbS_2_ transforms into the $\sqrt{3}\times \sqrt{3}$ ‐ phase (Figure 5b): the island height increased to 0.99 nm and the atomic resolution inset displays a $\left(\sqrt{3}\times \sqrt{3}\right)R30^{\circ }$ superstructure. Only a small portion remains as single‐layer NbS_2_, of which one piece is encircled. The island area fraction decreased from 0.36 to 0.28. This titration experiment allows one to calculate the amount of Nb per unit cell in the $\sqrt{3}\times \sqrt{3}$ ‐ phase. The total amount of Nb provided consists of 0.36 + 0.12 ML while the island area fraction is 0.28. Thus, each $\sqrt{3}\times \sqrt{3}$ ‐ phase unit cell contains $x=\frac{0.36+0.12}{0.28}$ or *x* = 1.71 Nb atoms within the limits of error. It appears likely that *x* = 5/3 due to a full Nb layer and an additional 2/3 Nb layer. Provided the 1/3 vacancies order, they could give rise to the $\sqrt{3}\times \sqrt{3}$ superstructure of the $\sqrt{3}\times \sqrt{3}$ ‐ phase.

**Figure 6:**
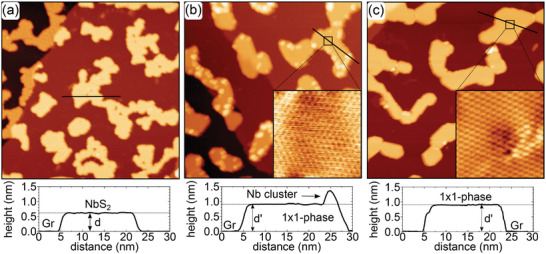
Formation of the 1 × 1 ‐ phase by Nb supply. a) Single‐layer NbS_2_ grown by deposition of 0.33 ML Nb in S background pressure at room temperature and annealed to 820 K in the absence of additional S supply. b) Sample after deposition of additional 0.33 ML Nb at 820 K. Inset: atomic resolution topograph of the boxed area. c) Sample after additional annealing to 1020 K. Inset: atomic resolution STM topograph of the boxed area. Height profiles are taken along the black lines in the topographs. Image information: (a) size 100 nm × 100 nm, *V*_s_ = 1.0 V, *I*_t_ = 0.23 nA; (b) size 100 nm × 100 nm, *V*_s_ = 1.0 V, *I*_t_ = 0.3 nA; Inset: 5 nm × 5 nm, *V*_s_ = 0.1 V, *I*_t_ = 5 nA; (c) size 100 nm × 100 nm, *V*_s_ = 1.2 V, *I*_t_ = 0.3 nA; Inset: 5 nm × 5 nm, *V*_s_ = 0.1 V, *I*_t_ = 5 nA.

Similarly, we deposited 0.33 ML Nb at 820 K on pre‐grown pristine single‐layer NbS_2_ islands with an area fraction of 0.33 (**Figure** **6**a). As apparent from Figure 6b, upon deposition the NbS_2_ islands transformed to the 1 × 1 ‐ phase: the island height increased to 0.93 nm and the atomic resolution inset displays a 1 × 1 structure with low corrugation. Additionally, small clusters are present at the island edges. The island area fraction marginally decreased from 0.33 to 0.29. With the same approach as above, one obtains formally an Nb content of 2.3 atoms per 1 × 1 ‐ phase unit cell. We tentatively conclude that a 1 × 1 ‐ phase unit cell contains 2 Nb atoms while the excess Nb is contained in the metallic clusters. Note that the clusters formed via this process differ from the ones observed after complete NbS_2_ dissociation at 1320 K annealing (compare Figure 2f), the clusters observed here are the result of still unreacted Nb, as dissociation is not expected to happen at 820 K. The sample remains in the 1 × 1 ‐ phase upon additional annealing to 1020 K while the clusters at the island edges largely disappear (compare Figure 6c). Their disappearance is presumably due to Nb already escaping under Gr at 1020 K.

It is remarkable that the $\sqrt{3}\times \sqrt{3}$ ‐ phase and the 1 × 1 ‐ phase form phase pure at 820 K when excess Nb is supplied, whereas plain annealing requires much higher temperatures of 970 and 1170 K, respectively, as seen by XPS (compare Figure 4a). The temperature difference to achieve the new phases between the two sets of experiments indicates that the kinetically difficult process in phase formation during annealing under sulfur‐poor conditions is the S dissociation and detachment to meet the progressively lower S content of the $\sqrt{3}\times \sqrt{3}$ ‐ phase and the 1 × 1 ‐ phase. The Nb deposition reliefs the need for S dissociation in order to achieve Nb excess. Given the presence of excess Nb, the reorganization of bonding to create the $\sqrt{3}\times \sqrt{3}$ ‐ phase and the 1 × 1 ‐ phase is apparently facile already at 820 K. Atomistic insight in how S detaches from the islands and how the islands reorganize into new phases is highly desirable, but would presumably require atomically resolved measurements at the temperature of phase reorganization ‐ a formidable task beyond the scope of the present manuscript.

**Figure 7:**
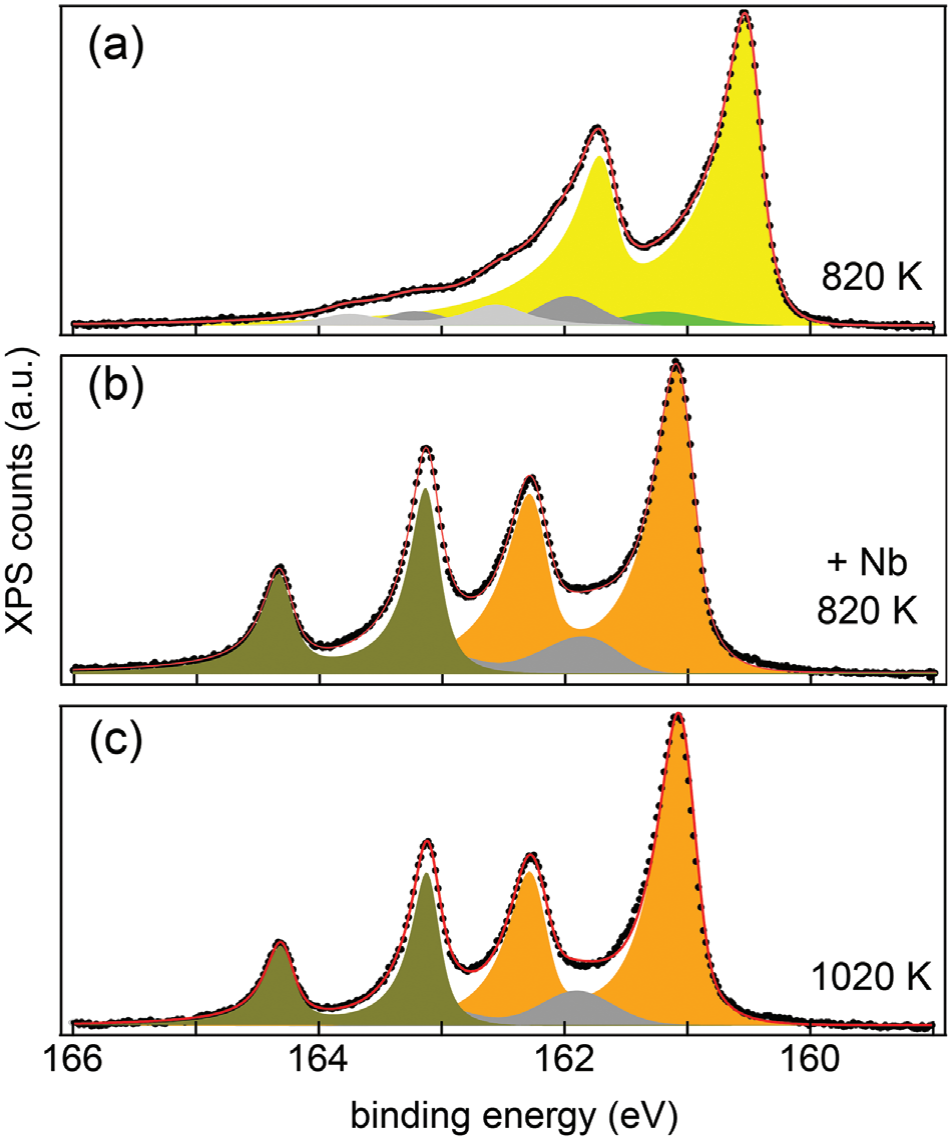
a) High‐resolution XPS of the S 2p core level of NbS_2_ on Gr/Ir(111). b) After deposition of additional Nb at 820 K. c) After annealing at 1020 K. Fit of each spectrum with five S 2p components.

XPS corroborates the transformation of NbS_2_ to the 1 × 1 ‐ phase using the same conditions as for the STM sequence presented in Figure 6. **Figure** **7**a shows the typical S 2p spectrum for pristine NbS_2_, similar to Figure 4b. After deposition of Nb at 820 K, the spectrum in Figure 7b is nearly identical to the spectrum obtained after annealing to 1220 K in the absence of Nb supply (compare Figure 4d) being characteristic of the 1 × 1 ‐ phase. Upon further annealing to 1020 K, the 1 × 1 ‐ phase remains unchanged and the related spectrum in Figure 7c is indistinguishable from the one obtained after 1 × 1 ‐ phase  formation at 1220 K without additional Nb supply shown in Figure 4d. The absence of S_NbS2_ and $S_{\mathrm{top}-\sqrt{3}}$ components in Figure 7b,c demonstrates that the Nb deposition at 820 K, i.e., below *T*_diss_, creates pure 1 × 1 ‐ phase and validates our method.

### 2.4 DFT Calculations

With the experimental information at hand and the help of DFT calculations we determined the structure of the 2D materials resulting from phase transformations of NbS_2_. We start the analysis with the 1 × 1 ‐ phase.

The 1 × 1 ‐ phase has the following properties: i) hexagonal symmetry and lattice parameter identical to single‐layer NbS_2_ within the limits of error; ii) it contains a smaller fraction of sulfur than NbS_2_ and even less than the $\sqrt{3}\times \sqrt{3}$ ‐ phase, since it evolves upon annealing from these phases under sulfur‐deficient conditions, accompanied by a gradual decrease of the S 2p intensity; iii) apparent height 0.93 nm, larger by 0.31 nm compared to NbS_2_; iv) 2 Nb atoms per unit cell; v) no superstructure; vi) only physisorbed to Gr.

It is well known from the Nb–S phase diagram and previous reports^[^ ^21^, ^22^ ^]^ that at high temperatures in bulk a NiAs‐type structure of NbS forms, with almost identical lattice parameter as NbS_2_. The NiAs structure is hexagonal, with As atoms forming a hexagonal close‐packed lattice, with Ni in octahedral sites, resulting in alternating atomic planes of Ni and As. Since the 1 × 1 ‐ phase contains two Nb atoms per unit cell, a natural starting point for the DFT calculations was just the NbS bulk unit cell, i.e., Nb_2_S_2_‐2D.

**Figure 8:**
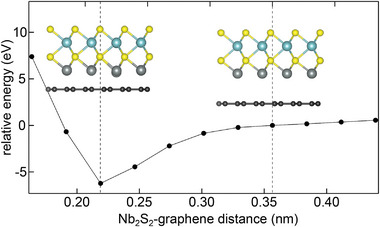
Chemisorption of Nb_2_S_2_‐2D to Gr. Relative total energy of minimum energy configuration of Nb_2_S_2_‐2D (lowest energy structure) as a function of the distance to Gr. Zero point of the energy scale is at 0.36 nm in the physisorbed state. Inset: side view ball models of relaxed DFT geometries for Nb_2_S_2_‐2D in the 0.36 and 0.22 nm Nb‐C distances. Nb atoms: cadet blue balls and gray; S atoms: yellow; dark gray: C atoms.

The minimum energy configuration of Nb_2_S_2_‐2D on Gr is surprisingly not of NiAs‐type. It consists of two Nb layers in trigonal prismatic coordination with the S atoms as well as the Nb atoms of the respective layers sitting atop each other, as shown in the ball model insets of **Figure** **8**. For higher energy structures compare Table S1 (Supporting Information). The ground state configuration (i.e., the lowest energy structure) is chemisorbed to Gr with Nb−plane to Gr distance of only 0.22 nm. However, our calculations also identified a local minimum configuration in which the Nb_2_S_2_‐2D layer is physisorbed at a distance of 0.36 nm. Furthermore, starting from the chemisorbed configuration and rigidly lifting the Nb_2_S_2_‐2D above the Gr we evaluated the total energy of the system at specific distances along the *z* −direction. Figure 8 shows the total slab energy versus distance. No significant barrier exists between the physisorbed and the chemisorbed state. As an additional note, the chemisorption of hypothetical Nb_2_S_2_‐2D to Gr is not surprising, given the expected high reactivity of the bare Nb, i.e., a 4*d* metal) toward the C atoms of Gr. Considering the high temperatures used in our experiments, one expects that any potential barrier between physisorbed and chemisorbed state can be overcome. Therefore Nb_2_S_2_‐2D should be chemisorbed to Gr. Given that the 1 × 1 ‐ phase islands are easy to move with the STM tip on Gr (see Figure S3b, Supporting Information] and that the C 1s core level is not affected during the phase transformations of physisorbed NbS_2_ (compare 8a, Supporting Information], the 1 × 1 ‐ phase is not chemisorbed. Thus, we can clearly exclude that the Nb_2_S_2_‐2D is observed in our experiments, regardless of the stacking sequence.

**Figure 9:**
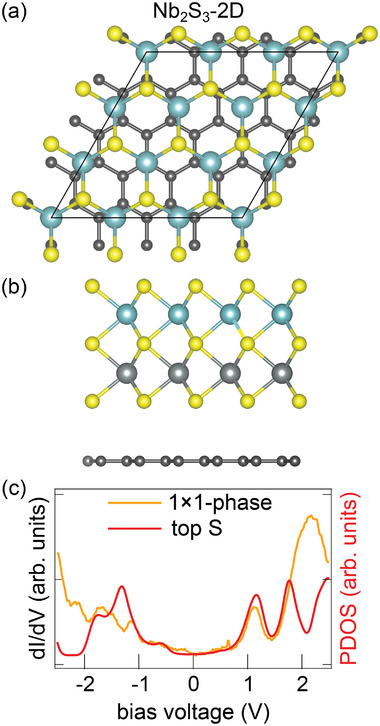
DFT calculated ball model representation of the 1 × 1 ‐ phase with stoichiometry Nb_2_S_3_‐2D in a) top and b) side view. Nb atoms: cadet blue balls and gray; S atoms: yellow; dark gray: C atoms. c) Differential conductance spectrum of the 1 × 1 ‐ phase (orange) compared to the DFT calculated partial density of states (PDOS) of the topmost S atoms (red). Spectrum parameters are *V*_stab_ = 2.5 V, *I*_stab_ = 0.7 nA, *V*_mod_ = 10 mV, *f*_mod_ = 811 Hz, *T*_s_ = 1.7 K.

As chemisorption has to be ruled out, we are forced to assume the presence of an additional passivating S layer, i.e., the 1 × 1 ‐ phase to be Nb_2_S_3_‐2D. Using DFT, all possible eight stacking sequences for Nb_2_S_3_‐2D were calculated (compare Table S2, Supporting Information). Irrespective of the stacking, Nb_2_S_3_‐2D is solely physisorbed to Gr, consistent with complete Nb passivation by S. The lowest energy structure is presented in **Figure** **9**a,b as top and side view ball model. It is again not the expected NiAs‐type structure, but displays all Nb atoms in trigonal prismatic coordination with the S and Nb atoms of the respective layers sitting atop each other. The structure possesses no reconstruction, as required. It has a lattice parameter of *a* = 0.333 nm in decent agreement with the experimental value of 0.330 nm. The calculated height of 0.976 nm matches reasonably well with the STM measured apparent heights of 0.93 nm at +1.00 V and 0.94 nm at −1.00 V. Figure 9c shows good agreement between the calculated top‐layer S partial density of states with a large‐range differential conductance spectrum.

The interpretation of the S 2p core level components is now straightforward given the Nb_2_S_3_‐2D stoichiometry: The S_btw_ component at 163.13 eV strongly shifted by 2.48 eV with respect to S_NbS2_ arises from S atoms located between two Nb planes, which provides a more electropositive environment compared to S atoms with Nb neighbors on only one side. This explains the significant binding energy shift, indicating a substantially altered chemical environment. The S_top‐1_ component at 161.05 eV shifted by 0.45 eV with respect to S_NbS2_ corresponds to the S top plane of atoms, while the bottom S atoms, with two Nb and two S atomic planes above, do not contribute significantly to the intensity due to substantial damping. The bottom S intensity is presumably hidden in the S_top‐1_ component.

The $\sqrt{3}\times \sqrt{3}$ ‐ phase is quite similar to the 1 × 1 ‐ phase in terms of symmetry, lattice parameter, apparent height (0.99 nm), and the overall shape of the S 2p core‐level spectra. However, it displays a $\sqrt{3}\times \sqrt{3}$ superstructure and contains only ≈5/3 Nb atoms per unit cell. From previous work^[^ ^21^ ^]^ it is known that in the NiAs‐type bulk structures of stoichiometry Nb_2 − *x*_S_2_, Nb vacancies are present in every second Nb layer.

For the $\sqrt{3}\times \sqrt{3}$ ‐ phase it is therefore most reasonable to assume that 1/3 of Nb is missing in one of the two Nb layers. This pattern would naturally give rise to the $\sqrt{3}\times \sqrt{3}$ superstructure, which is observed in LEED and STM. The stoichiometry of the $\sqrt{3}\times \sqrt{3}$ ‐ phase is consequently Nb_5/3_S_3_‐2D.

**Figure 10:**
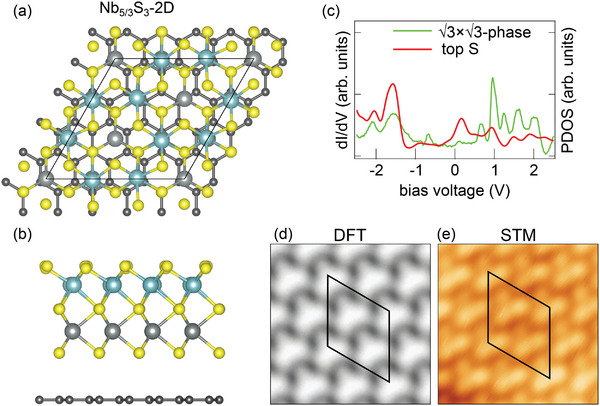
DFT calculated ball model representation of the $\sqrt{3}\times \sqrt{3}$ ‐ phase with stoichiometry Nb_5/3_S_3_‐2D in a) top and b) side view. Nb atoms: cadet blue balls and gray; S atoms: yellow; dark gray: C atoms. c) Differential conductance spectrum of the $\sqrt{3}\times \sqrt{3}$ ‐ phase (green) compared to the DFT calculated partial density of states (PDOS) of the topmost S atoms (red). d) DFT calculated STM topograph compared to e) measurement. Spectrum parameters: *V*_stab_ = 3 V, *I*_stab_ = 0.8 nA, *V*_mod_ = 10 mV, *f*_mod_ = 797 Hz, *T*_s_ = 1.7 K.

The lowest energy structure of Nb_5/3_S_3_‐2D is displayed in **Figure** **10**a,b as top and side view ball model (compare Table S3, Supporting Information for other calculated structures). Again, the minimum energy is not a NiAs‐type structure. While the Nb atoms in the complete atomic plane close to Gr are still in trigonal prismatic coordination, the top Nb plane with the regularly distributed Nb vacancies has the Nb atoms in octahedral coordination. The structure displays a $\sqrt{3}\times \sqrt{3}$ superstructure, as required. It has a lattice parameter of 0.333 nm, in agreement with the experimental value of 0.333 nm. The calculated height of 0.964 nm matches reasonably well with the average of the STM measured apparent heights of 0.99 nm at +1.00/ V and 0.90 nm at –1.00 V. Figure 10c shows decent agreement of the calculated top S partial density of states with a large‐range differential conductance spectrum, reproducing the number of peaks on the unoccupied region, but not their location nor intensity. Figure 10d,e shows excellent agreement between the DFT simulated STM topograph and the measurement.

The S 2p spectrum of the $\sqrt{3}\times \sqrt{3}$ ‐ phase is interpreted as follows: the $S_{\mathrm{top}-\sqrt{3}}$ component at 161.33 eV is assigned to top sulfur, while the S_btw_ component corresponds to the highly coordinated sulfur atoms between Nb atomic planes. The larger FWHM of both components for the $\sqrt{3}\times \sqrt{3}$ ‐ phase, compared to the 1 × 1 ‐ phase, indicates a less homogeneous state of sulfur, which due to the Nb vacancies, are bound to fewer Nb atoms on one side.

## 3 Conclusion and Outlook

In summary, under sulfur‐poor conditions and heating single‐layer NbS_2_ transforms to the more Nb‐rich compounds Nb_5/3_S_3_‐2D (970 K, $\sqrt{3}\times \sqrt{3}$ ‐ phase) and Nb_2_S_3_‐2D (1170 K, 1 × 1 ‐ phase). Nb_5/3_S_3_‐2D displays a $\sqrt{3}\times \sqrt{3}$ superstructure caused by regularly arranged Nb vacancies in the top Nb layer. The same compounds may also be created by deposition of excess Nb under sulfur‐poor conditions at 820 K, a temperature at which NbS_2_ does not show changes with time in the absence of a Nb flux. The compounds consist of two Nb layers sandwiched between three S layers and are inert, covalently bound 2D materials. Consequently, these compounds emerge from NbS_2_ through covalent transformation. As uncovered by density functional theory calculations, the layer stacking sequence is unique for each compound and can not be derived from corresponding bulk materials.

Here we demonstrated the transformation of a transition metal disulfide, a material that displays van der Waals gaps in bulk, into a covalently bound 2D‐material. It may be speculated that such a transformation is possible whenever a more metal‐rich compound is present in the bulk phase diagram. Since S is generally the more volatile component as compared to the metal, annealing at a suitable temperature can be expected to trigger such a phase transformation. Here we could demonstrate that metal supply can lower the temperature needed for the phase transformation substantially, as it lifts the condition of S loss to induce it. The phase transformation can be expected to proceed smoothly when the more metal‐rich covalently bound compound displays in its crystal structure a similar layered structure of alternating planes of sulfur and metal as the initial transition metal disulfide. This includes phases that are coined self‐intercalation compounds, where the van der Waals gap is partially filled with metal species, thereby shifting the stoichiometry to the metal‐rich side. Examples are Ta_*x*_S_*y*_^[^ ^8^ ^]^ and V_*x*_S_*y*_^[^ ^11^ ^]^ self‐intercalation compounds. When the monosulfide exists in the bulk phase diagram in a structure similar to NiAs (modulo stacking changes) the formation of covalently bound materials composed of 5 atomic planes – two full or partial metal layers separated and sandwiched by sulfur layers – appears likely. Thus similar covalently bound 2D‐materials are expected to be formed for Ti–S^[^ ^29^ ^]^ and Cr–S.^[^ ^30^, ^31^ ^]^ These new covalently bound 2D materials are yet to be explored. They can be expected to enrich our ability to design new electronic and magnetic functions with ultimately thin 2D materials.

## 4 Experimental Section

### Experimental Methods

The experiments were carried out in three ultrahigh vacuum systems (base pressure in low 10^−10^ mbar range). All systems were equipped with sample preparation and growth facilities as well as LEED. STM measurements were conducted in two systems in Cologne while XPS was performed at the FlexPES beamline end station at MAX IV Laboratory, Lund.

### Substrate Preparation

Ir(111) was cleaned by cycles of keV Ar^+^ or Xe^+^ sputtering and flash annealing to 1520 K. Gr was grown by ethylene exposure of Ir(111) to saturation at room temperature, subsequent flash annealing to 1470 K, and followed by exposure to ≈800 L of ethylene at 1370 K. As confirmed by STM and LEED a closed single crystal Gr monolayer on Ir(111) results.^[^ ^32^ ^]^

### Sample Preparation

Single‐layer H‐NbS_2_ was prepared by exposing Gr/Ir(111) to a flux of ≈6 × 10^15^ atoms per m^2^s Nb from an e‐beam evaporator in a background pressure of ≈8 × 10^−9^ mbar elemental S. The S was supplied by a pyrite filled Knudsen cell ≈10 cm away from the sample. Growth was conducted for 510 s at room temperature, followed by 360 s annealing at 820 K. During annealing, the Knudsen cell was turned off. However, since the S pressure decreases slowly, the S pressure remained non‐zero, albeit well below 8 × 10^−9^ mbar. Since H‐NbS_2_ growth takes place with excess S reevaporating, the amount of H‐NbS_2_ formed is characterized through the amount of Nb deposited. 1 monolayer (ML) of Nb corresponds to the Nb amount in a full single layer of NbS_2_, i.e., to 1.12 × 10^19^ atoms per m^2^. Phase transformations of H‐NbS_2_ resulted from deposition of elemental Nb onto single‐layer NbS_2_ at different temperatures, or by annealing to temperatures above 820 K, or both, as specified where the respective data is discussed.

### STM Measurements

The samples were investigated in situ by STM, either at 300 K or at 1.7 K after ultrahigh vacuum transfer into a bath cryostat. Scanning tunneling spectroscopy was conducted at 1.7 K with Au‐covered W tips calibrated using the surface state of Au(111).^[^ ^33^, ^34^ ^]^ Constant‐current STM topographs were recorded with sample bias *V*_s_ and tunneling current *I*_t_ specified in each figure. *dI*/*dV* spectra were recorded with stabilization bias *V*_stab_ and stabilization current *I*_stab_ using a lock‐in amplifier with a modulation frequency *f*_mod_ and modulation voltage *V*_mod_, also specified in the captions.

### XPS Measurements

The XPS experiments were conducted at the FlexPES beamline at MAX IV Laboratory, Lund, Sweden.^[^ ^35^ ^]^ The growth of Nb_*x*_S_*y*_‐2D compounds at the beamline was carried out with a Nb evaporator calibrated by STM in the home lab. High‐resolution XPS of core‐levels was performed in normal emission geometry with a spot size of 50 µm × 50 µm and at room temperature. The core levels were monitored with photon energies to maximize surface sensitivity: 150 eV for Ir 4f, 260 eV for S 2p, 380 eV for C 1s, 300 eV for Nb 3d. Overview spectra and high‐resolution O 1s spectra obtained at the first and last measurements of an annealing series confirmed that no other species were present. Curve fitting was performed with a pseudo‐Voigt function. The asymmetry is included by an energy‐dependent variation of the full‐width‐at‐half maximum. The width, asymmetry and ratio of Gaussian to Lorentzian contributions were fixed for each component, meaning that they were not allowed to vary between spectra taken at different annealing temperatures. The center energy of each component was granted a ±100 meV variation between different spectra while the intensities of the components were unconstrained.

### Theoretical Calculations

The spin‐polarized calculations were done by using DFT^[^ ^36^ ^]^ and the projector augmented plane wave method^[^ ^37^ ^]^ as implemented in the VASP code.^[^ ^38^, ^39^ ^]^ A 500 eV energy cutoff was used for the plane wave expansion of the Kohn‐Sham wave functions.^[^ ^40^ ^]^ To account for the nonlocal correlation effects like van der Waals interactions,^[^ ^41^ ^]^ all structural relaxations were done by using vdW‐DF2^[^ ^42^ ^]^ functional containing a revised Becke (B86b) exchange,^[^ ^43^, ^44^ ^]^ while the analysis of the electronic structure was performed by using the standard PBE exchange‐correlation energy functional.^[^ ^45^ ^]^

## Conflict of Interest

The authors declare no conflict of interest.

## Supporting information

Supporting Information

## Data Availability

The data that support the findings of this study are available from the corresponding author upon reasonable request.
