# Supplementary material for: Engineering 2D Materials from Single‐Layer NbS2
Source: Small. 2024 Nov 25;21(3):2408044. doi: 10.1002/smll.202408044 (PMC11753502; doi:10.1002/smll.202408044)
Supplement: Supplementary file 1 — Supporting Information [file SMLL-21-2408044-s001.pdf]

NANO · MICRO
small

Supporting Information

for *Small*, DOI 10.1002/smll.202408044

Engineering 2D Materials from Single-Layer NbS_2

*Timo Knispel, Daniela Mohrenstecher, Carsten Speckmann, Affan Safeer, Camiel van Efferen, Virgínia Boix, Alexander Grüneis, Wouter Jolie, Alexei Preobrajenski, Jan Knudsen, Nicolae Atodiresei, Thomas Michely and Jeison Fischer**

Supplementary Information: Engineering two-dimensional materials from single-layer NbS_2

Timo Knispel,[1] Daniela Mohrenstecher,[1] Carsten Speckmann,[1] Affan Safeer,[1] Camiel van Efferen,[1] Virgínia Boix,[2] Alexander Grüneis,[1] Wouter Jolie,[1] Alexei Preobrajenski,[3] Jan Knudsen,[2,3] Nicolae Atodiresei,[4] Thomas Michely,[1] and Jeison Fischer[1,*]

[1]*II. Physikalisches Institut, Universität zu Köln, Zülpicher Straße 77, D-50937 Köln, Germany*

[2]*NanoLund and Division of Synchrotron Radiation Research, Department of Physics, Lund University, SE-221 00 Lund, Sweden*

[3]*MAX IV Laboratory, Lund University, SE-221 00 Lund, Sweden*

[4]*Peter Grünberg Institut (PG-1), Forschungszentrum Jülich, Wilhelm-Johnen-Straße, D-52428 Jülich, Germany*

(Dated: November 15, 2024)

[*] Corresponding author: jfischer@ph2.uni-koeln.de

CONTENTS

Supplementary Note 1: NbS_2 bilayer islands after room temperature growth and annealing at 820 K 3
Supplementary Note 2: Nb intercalation after 1120 K annealing 4
Supplementary Note 3: Nb_xS_y islands displaced by the STM tip 5
Supplementary Note 4: Additional annealing sequence 6
Supplementary Note 5: Nb 3d core-level as a function of temperature during NbS_2 transformation by annealing. 7
Supplementary Note 6: LEED of the $\sqrt{3} \times \sqrt{3}$ - phase 8
Supplementary Note 7: S 2p components and total S 2p core level intensity as a function of temperature during NbS_2 transformation by annealing. 9
Supplementary Note 8: C 1s and Ir 4f core-levels as a function of temperature during NbS_2 transformation by annealing. 10
Supplementary Note 9: DFT calculated Nb_2S_2-2D structures on Gr 11
Supplementary Note 10: DFT calculated Nb_2S_3-2D structures on Gr 12
Supplementary Note 11: DFT calculated $Nb_{5/3}S_3$-2D structures on Gr 13

Supplementary Note 1: NbS_2 bilayer islands after room temperature growth and annealing at 820 K

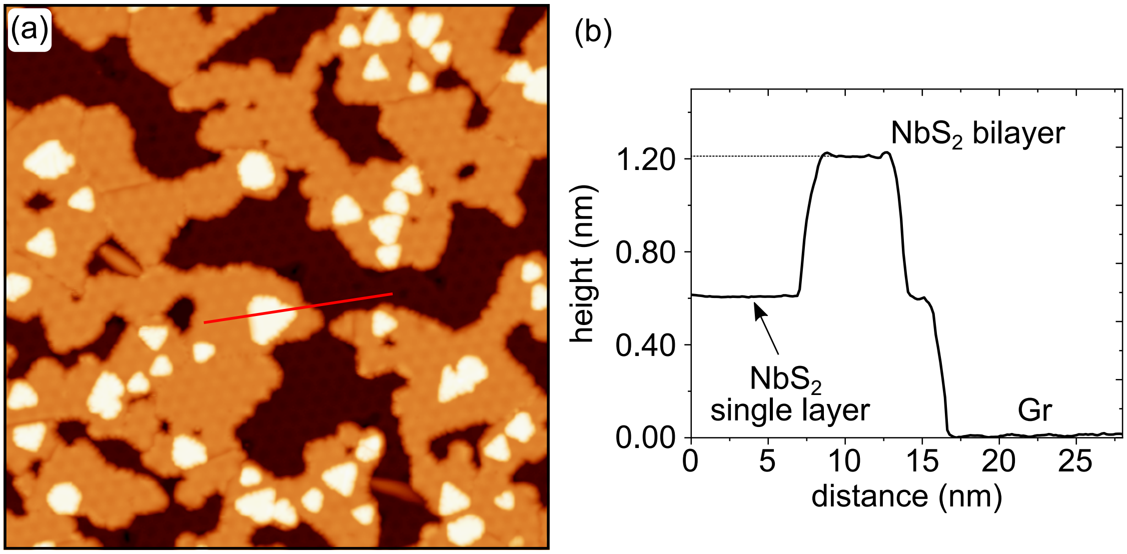

Figure S1. NbS_2 single layer and bilayer islands after growth on Gr/Ir(111) and annealing to 820 K. (a) STM topograph. (b) Height profile measured along the red line in (a). Image information: size 75 nm × 75 nm, $V_s = 1.0$ V, $I_t = 0.1$ nA, $T_s = 0.4$ K

Supplementary Note 2: Nb intercalation after 1120 K annealing

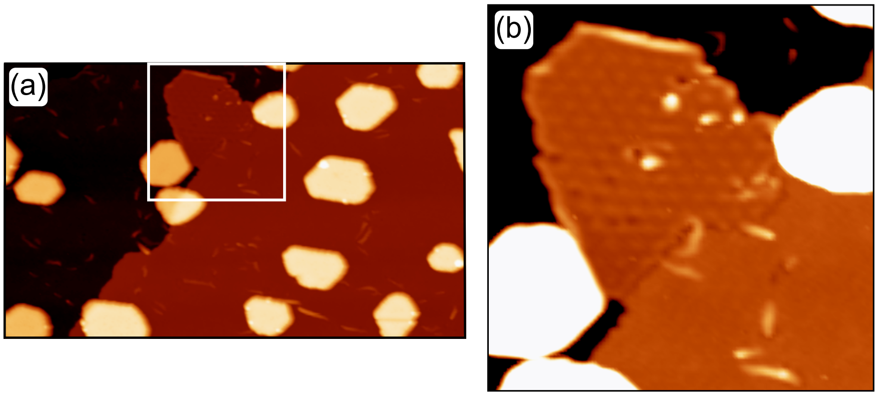

Figure S2. (a) Same STM topograph as in Figure 2(d). (b) Contrast-enhanced zoom of boxed area in (a). The peninsula has a stronger moiré contrast than the rest of the substrate. This indicates the presence of a metal underneath Gr that interacts stronger with Gr than Ir does. Defects in Gr are also visible in that area. We tentatively assume that the peninsula is formed by Nb stemming from Nb_xS_y decomposition during annealing that penetrated the Gr layer through the point defects and intercalated.

Supplementary Note 3: Nb_xS_y islands displaced by the STM tip

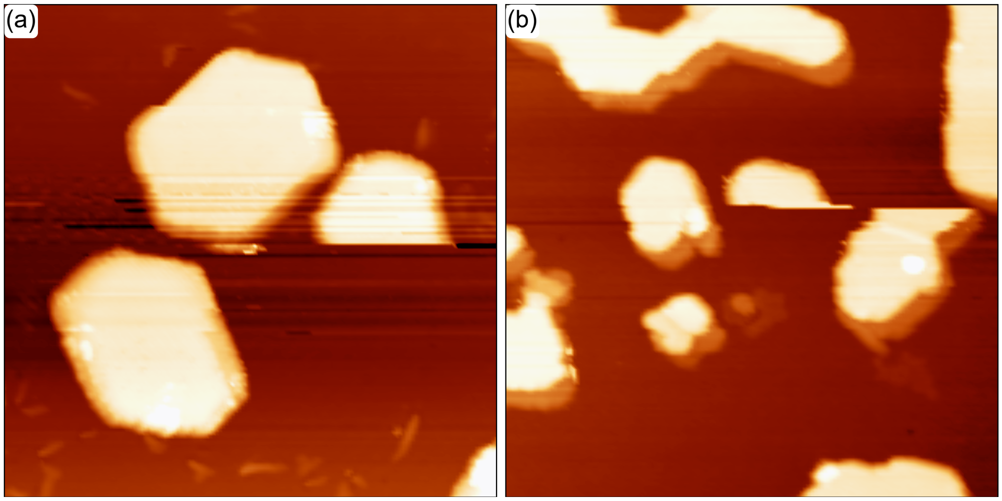

Figure S3. (a) $\sqrt{3} \times \sqrt{3}$ - phase and (b) 1×1 - phase islands displaced by the STM tip under standard imaging conditions. Abrupt horizontal island cuts indicate displacement. The STM tip induced shift of both types of islands demonstrates their weak coupling to the substrate. Image information: (a,b) size $60\,\text{nm}\times 60\,\text{nm}$, (d) $V_s = 0.92\,\text{V}$, $I_t = 0.33\,\text{nA}$.

Supplementary Note 4: Additional annealing sequence

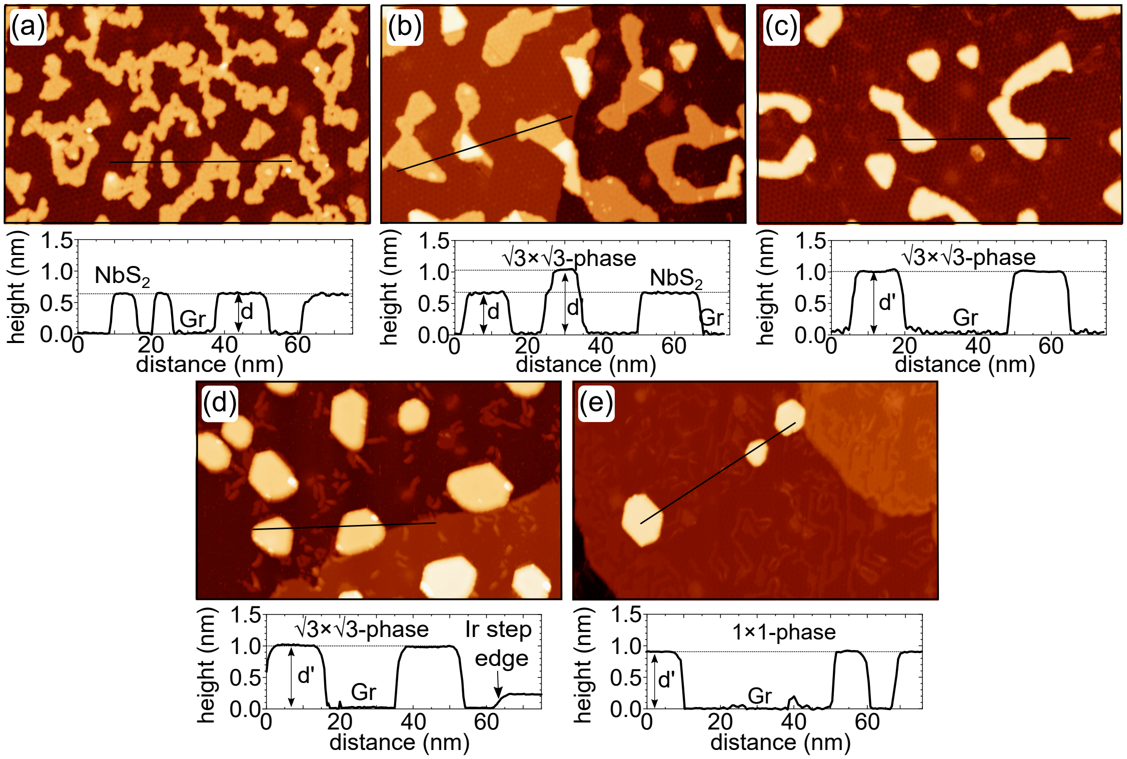

Figure S4. STM topographs of an additional independent isochronal annealing sequence of initial single-layer NbS_2 islands without supply of additional S. Annealing intervals are 360 s. (a) single-layer NbS_2 islands after room temperature growth and annealing to 820 K. (b)-(f) After additional annealing to (b) 920 K, (c) 1020 K, (d) 1120 K, and (e) 1220 K. Height profiles along the black lines are shown below the topographs. Height levels $d = 0.62$ nm, $d' = 0.99$ nm and $d' = 0.93$ nm distinguish between single-layer NbS_2, $\sqrt{3} \times \sqrt{3}$ - phase, 1×1 - phase, respectively. Image information: for all size 150 nm × 90 nm, a V_s =1.0 V, $I_t = 0.23$ nA; (b) $V_s = 0.95$ V, $I_t = 0.34$ nA; (c) $V_s = 1.0$ V, $I_t = 0.26$ nA; (d) $V_s = 0.92$ V, $I_t = 0.33$ nA; (e) $V_s = 1.0$ V, $I_t = 0.32$ nA.

Supplementary Note 5: Nb 3d core-level as a function of temperature during NbS_2 transformation by annealing.

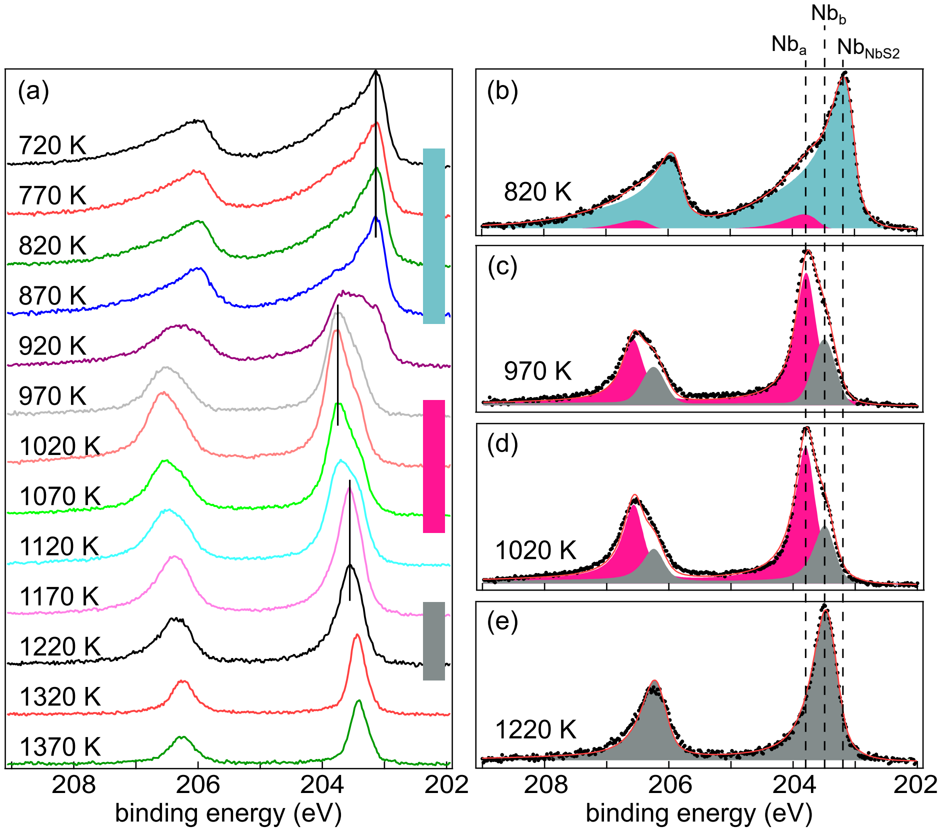

Figure S5. (a) High-resolution X-ray photoemission spectroscopy of the Nb 3d core levels during NbS_2 transformation by annealing from 720 K to 1370 K. Each data point was measured at room temperature after annealing to the indicated temperature without supply of addition S. The spectra are grouped in three temperature ranges according to their similarities: cadet blue, magenta, and gray. (b)-(d) Nb 3d core level spectra after annealing to (b) 820 K, (c) 970 K, (d) 1020 K, and (e) 1220 K fitted with components NbS_2, Nb_a, and Nb_b. After annealing to 820 K Nb 3d shows in (b) a single component NbS_2 at 203.34 eV attributed to Nb in NbS_2. After annealing to 970 K and 1020 K (in (c) and (d) we find two Nb components Nb_a at 203.82 eV and Nb_b at 203.51 eV for the Nb in $Nb_{5/3}S_3$-2D. The Nb in the partially occupied Nb plane with Nb vacancies is attributed to Nb_a, Nb in the fully occupied Nb bottom plane to Nb_b. Finally, after annealing to 1220 K in (e), the Nb_a component disappears and a single component spectrum is restored, since in Nb_2S_3-2D all Nb atoms are again in an identical chemical environment. Photon energy: $h\nu = 300$ eV for Nb 3d.

Supplementary Note 6: LEED of the $\sqrt{3} \times \sqrt{3}$ - phase

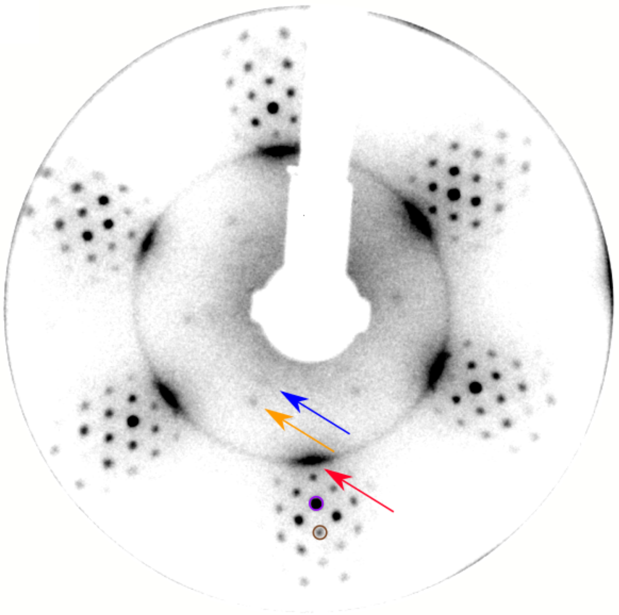

Figure S6. Inverted 140 eV LEED pattern of the $\sqrt{3} \times \sqrt{3}$ - phase obtained after annealing NbS_2 to 1020 K. First order reflections of Ir and Gr are marked with magenta and brown circles, respectively. A fundamental spot of the $\sqrt{3} \times \sqrt{3}$ - phase lattice being identical to the initial NbS_2 lattice is marked by a red arrow. Two sets of ($\sqrt{3} \times \sqrt{3}$ R)30° superstructure spots are distinguishable. One is with respect to Ir and due to S intercalated between Ir(111) and Gr (green arrow). The other set is with respect to the fundamental $\sqrt{3} \times \sqrt{3}$ - phase lattice reflection (blue arrow).

Supplementary Note 7: S 2p components and total S 2p core level intensity as a function of temperature during NbS_2 transformation by annealing.

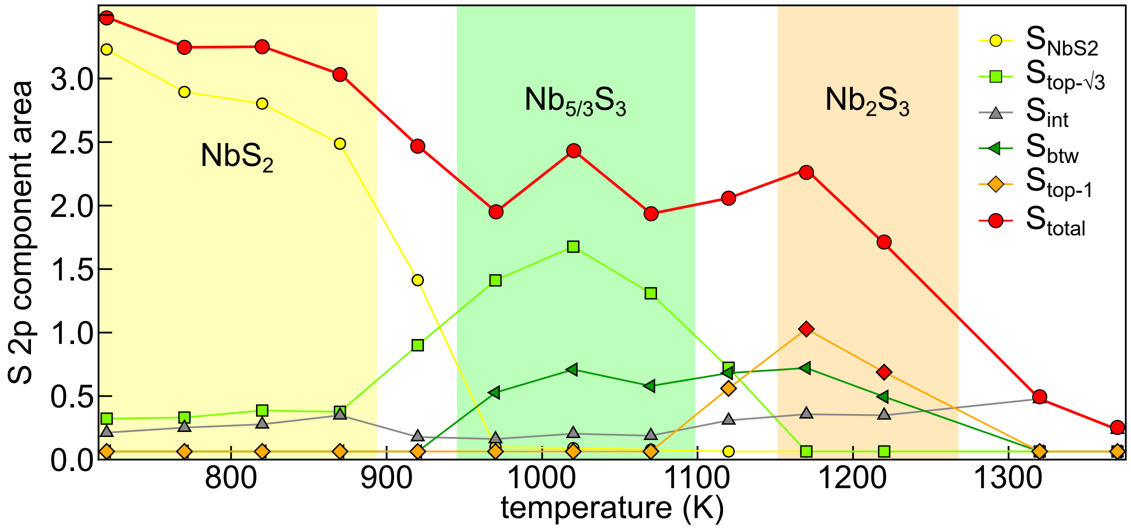

Figure S7. Integrated intensities of the fit components (as in Figure 4(b-d) in the main text) as a function of temperature. Total S 2p intensity in red. The background is color coded according to the dominating S 2p component: yellow (NbS_2), green ($\sqrt{3} \times \sqrt{3}$ - phase), and orange (1×1 - phase).

Supplementary Note 8: C 1s and Ir 4f core-levels as a function of temperature during NbS_2 transformation by annealing.

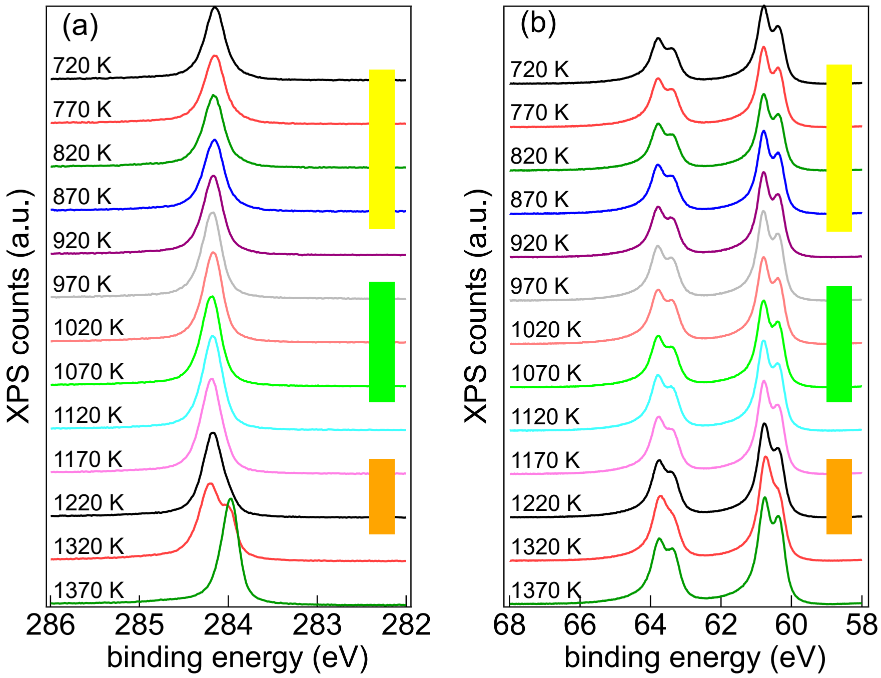

Figure S8. High-resolution X-ray photoemission spectroscopy of the C 1s (a) and Ir 4f (b) core levels during NbS_2 transformation by annealing from 720 K to 1370 K. Each data point was measured at room temperature after annealing to the indicated temperature without supply of addition S. Three temperature ranges are grouped according to the features identified in the S 2p core level from Figure 4(a) in the main text. The groups are color coded accordingly: yellow, green, and orange. Photon energies: $h\nu = 380$ eV for C 1s, and $h\nu = 150$ eV for Ir 4f.

Supplementary Note 9: DFT calculated Nb_2S_2-2D structures on Gr

Nb coordination	1H	1H	1T	1T	1H	1H
Nb-Nb position	shifted	shifted	aligned	aligned	aligned	aligned
structure	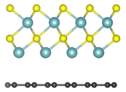	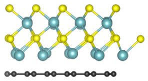	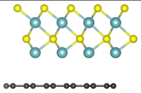	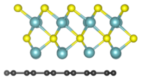	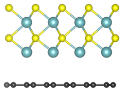	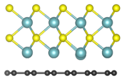
ΔE (eV/Nb_2S_2)	1.552	0.719	0.952	0.198	0.766	0.0000
Nb-C bond	phys	chem	phys	chem	phys	chem
height (nm)	0.827	0.702	0.833	0.699	0.826	0.699
trimerization	1×1	yes	1×1	yes	1×1	1×1

Table S1. DFT calculated Nb_2S_2-2D structures on Gr. Nb coordination is either trigonal prismatic (H) or octahedral (T)

Supplementary Note 10: DFT calculated Nb_2S_3-2D structures on Gr

coordination	1T/1T	1H/1T	1T/1H	1H/1H
Nb-Nb position	aligned	aligned	aligned	aligned
structure	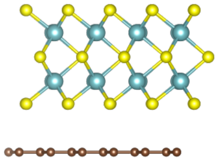	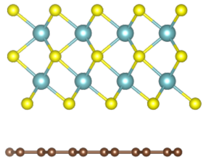	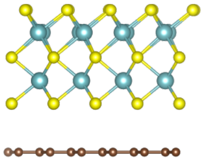	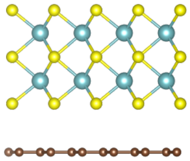
ΔE (eV/Nb_2S_3)	0.4538	0.3049	0.2634	0.0000
height (nm)	0.975	0.975	0.975	0.976
trimerization	yes	1×1	yes	1×1
coordination	1T/1T	1T/1H	1H/1T	1H/1H
Nb-Nb position	shifted	shifted	shifted	shifted
structure	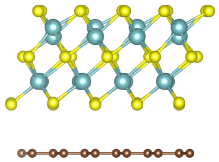	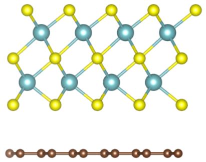	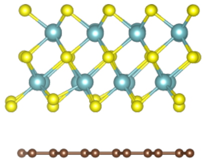	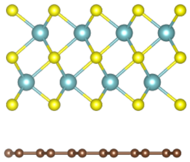
ΔE (eV/Nb_2S_3)	1.3786	1.0962	1.0517	0.6858
height (nm)	0.977	0.984	0.983	0.984
trimerization	yes	1×1	yes	1×1

Table S2. DFT calculated Nb_2S_3-2D structures on Gr for all possible stacking sequences. Nb coordination is either trigonal prismatic (H) or octahedral (T)

Supplementary Note 11: DFT calculated $Nb_{5/3}S_3$-2D structures on Gr

coordination	1T/1H		1H/1H		1T/1T	
Nb-Nb position	aligned		aligned		aligned	
1/3 Nb missing	top	bottom	top	bottom	top	bottom
structure	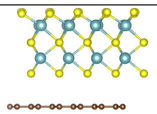	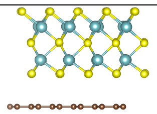	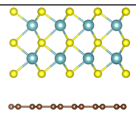	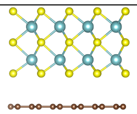	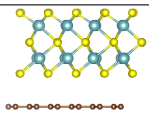	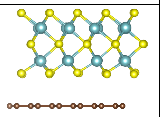
height (nm)	0.964	0.970	0.968	0.972	0.958	0.954
ΔE (eV/$Nb_{5/3}S_3$)	0.0000	0.6011	0.2378	0.2505	0.3736	0.3698

Table S3. Calculated $Nb_{5/3}S_3$-2D structures on Gr. Nb coordination is either trigonal prismatic (H) or octahedral (T)
